# Protein Surface Interactions—Theoretical and Experimental Studies

**DOI:** 10.3389/fmolb.2021.706002

**Published:** 2021-07-09

**Authors:** Fabio C. L. Almeida, Karoline Sanches, Ramon Pinheiro-Aguiar, Vitor S. Almeida, Icaro P. Caruso

**Affiliations:** ^1^ Institute of Medical Biochemistry—IBqM, Federal University of Rio de Janeiro, Rio de Janeiro, Brazil; ^2^ National Center for Structural Biology and Bioimaging (CENABIO)/National Center for Nuclear Magnetic Resonance (CNRMN), Federal University of Rio de Janeiro, Rio de Janeiro, Brazil; ^3^ Multiuser Center for Biomolecular Innovation (CMIB), Institute of Biosciences, Letters and Exact Sciences (IBILCE), São Paulo State University “Júlio de Mesquita Filho” (UNESP), São Paulo, Brazil

**Keywords:** surface, solvation, clusters, interdomain, NMR, dynamics, hydrophobic surface clusters

## Abstract

In this review, we briefly describe a theoretical discussion of protein folding, presenting the relative contribution of the hydrophobic effect versus the stabilization of proteins *via* direct surface forces that sometimes may be overlooked. We present NMR-based studies showing the stability of proteins lacking a hydrophobic core which in turn present hydrophobic surface clusters, such as plant defensins. Protein dynamics measurements by NMR are the key feature to understand these dynamic surface clusters. We contextualize the measurement of protein dynamics by nuclear relaxation and the information available at protein surfaces and water cavities. We also discuss the presence of hydrophobic surface clusters in multidomain proteins and their participation in transient interactions which may regulate the function of these proteins. In the end, we discuss how surface interaction regulates the reactivity of certain protein post-translational modifications, such as S-nitrosation.

## Introduction

Since the Anfinsen (Anfinsen, 1972) discovery that after chemical denaturation proteins can be refolded *in vitro*, the thermodynamic hypothesis became the most accepted model for protein folding. The question of what are the major forces that stabilize the protein and control the folding pathway arose as fundamental. The balance between hydrophobicity and hydrophilicity is crucial for protein folding and also for its structural property. This balance is known to be essential for the folding of globular proteins. While in intrinsically disordered proteins (IDPs) the balance is shifted toward the hydrophilicity, displaying an excess of hydrophilic residues, for globular proteins, there is a hydrophobic collapse mediated by a solvent entropic effect. The entropy increases significantly when a hydrophobic solute is transferred from an aqueous solution to a nonpolar environment, such as the protein core. The explanation (Kauzmann, 1959; Baldwin and Rose, 2016) for the entropy penalty is in the Gibbs free energy (ΔG°), enthalpy (ΔH°), and entropy (ΔS°) change for the hydration of hydrophobic solutes. For aqueous alkane, the enthalpic contribution is negative and favorable, while the entropic contribution is largely negative and unfavorable. The original explanation was on the rigidity of hydrocarbon hydration shells made of clathrate water (Kauzmann, 1959). New experiments suggest that the hydration shell made of clathrate water does not explain completely the entropic penalty. The best description is a hydration shell formed by Van der Waals (VDW) attraction, which also leads to increased rigidity of the water molecules (Baldwin, 2014; Baldwin and Rose, 2016). Until now we poorly understand the solvation of hydrophobic residues. Baldwin and Rose state that this limitation precludes our ability to predict the free-energy values that can drive the folding process (Baldwin and Rose, 2016). In this review, we do not intend to deeply discuss the physical chemistry of protein solvation. We start analyzing the importance of solvation in proteins without a typical hydrophobic core (named core-less proteins) (Machado et al., 2018a; Pinheiro-Aguiar et al., 2020). These proteins form hydrophobic clusters at the surface that need to be better understood. They are an unexplored avenue to the understanding of the solvation of exposed hydrophobic residues. We extend the analysis showing that the surface hydrophobic clusters are present in globular proteins and have an important impact on transient interactions in multi-domain proteins (Pinheiro et al., 2019) and may have an impact on the reactivity of residues participating in post-translational modifications.

Proteins are large molecules, imposing an intrinsic large number of degrees of freedom on the polypeptide chain and an astronomical number of possible conformations. If a protein had to sequentially sample among all possible conformations, it would take a time longer than the age of the Universe to access its native folded conformation. The Levinthal paradox (Levin, 2004) is based on the fact that, despite all that, proteins fold spontaneously on milliseconds to seconds time-scale. Computational calculations show that polypeptide chains evolved to reach the minimum energy conformational state by shaping kinetic favorable pathways in the energy landscape (Bryngelson et al., 1995; Ferreiro et al., 2014). The evolutionary shaping of the energy funnel is mediated by the balance of forces at each conformational state, in the local and global minima and its transition states (Wolynes, 2015).

As described earlier, water plays an essential role in determining protein stability and the kinetic folding pathways. It is believed that hydrophobic collapse is the dominant effect that drives protein folding. Nevertheless, hydrophilic residues have also an important contribution to protein stability and folding pathways (Durell and Ben-Naim, 2017). It is well known that waters interact with proteins in different regimes. While bulk water has high degrees of freedom when compared to the freedom of protein dihedrals, there are tightly bound waters, which bind to the protein as if it were part of it, having similar freedom as the protein chains. There is an intermediate regime, with water molecules that are part of the solvation shell, which interacts transiently with the protein surface contributing to the protein stability and modulating the interaction of protein itself or other ligands (Fernández and Scheraga, 2003; Papoian et al., 2003). Water molecules have been shown to participate in protein-protein interaction, bridging the protein interfaces.

The solvation of the hydrophilic and hydrophobic residues involved in the protein surface forces is the main topic of this review. (Ben Naim, 2013; Durell and Ben-Naim, 2017) used computational simulations to predict the attractive and repulsive potential of mean force (PMF) of the hydrophilic and hydrophobic interactions that contribute to protein folding. Their data suggest that hydrophilic surface forces may have an underestimated contribution to the overall structure stabilization. First, they considered the forces and potentials in nonpolar solutes, demonstrating that the interactions between hydrophobic solutes in water are mediated by direct and solvent-induced forces. Repulsive forces occur because of the steric reorganization and disruption of the water molecules around the solute. Attractive direct and solvent induced PMF contribute to the interaction of two hydrophobic molecules in water. The simulations suggest that the solvent-induced attractive force exerts a higher contribution than the direct forces for methane-methane stabilization. For larger alkanes, the solute induced PMF contributes approximately equally to the direct force. In all simulated situations, the water solvation of hydrophobic molecules contributes significantly to the total attractive force.

On the surface of proteins and other biomolecules, the role of the solvent-induced attractive force may be underestimated. Feng and colleagues (Feng et al., 2019) demonstrated the importance of this effect by investigating the effect of the hydrophobic interaction on the stability of the DNA duplex. They measured the effect of polyethylene glycol 400 (PEG400) in the base-pairing stability. Using atomic force microscopy, they verified a decrease in the DNA stretching force due to a decrease in base-stacking energy caused by transient fluctuations of the bases. The fluctuations, referred to as longitudinal breathing, involve the disruption of the hydration shell of the DNA, leading to increased hydration in the interior of the DNA and a consequent decrease in hydrogen bond energy. In the presence of abundant water, the intact hydration shell promotes a solvent-induced hydrophobic interaction among the nitrogenous bases, the DNA interior gets dryer, allowing hydrogen bonds to occur in an ideal geometry. This important finding reveals that, instead of the base-pairing hydrogen bonds being the main responsible for the DNA stabilization, it involves the coin-pile stacking of base pairs due to the hydrophobic effect. The hydration shell shields the DNA, making its interior dry. The disruption of the hydration shell decreases the solvent-induced hydrophobic forces among the bases, causing fluctuation of the bases, promoting the formation of holes in the DNA interior and a consequent weakening of the Watson-Crick hydrogen bonds.

For hydrophilic groups interacting in an aqueous solution, the solvent-induced forces contribute even more significantly. For the simulation with two hydroxyls, the direct force is repulsive while the solvent-induced force is attractive by the formation of a water molecule bridging the two hydroxyls. For the simulations with 3 and 4 hydroxyls, the solvent-induced attractive force is even bigger, and the direct forces are also attractive. In the surface of a globular protein, the probability of finding clusters of hydrophilic side chains is high, and thus there is a good chance of having water molecules playing the role of bridging side-chains, contributing significantly to the stabilization of the overall fold. In bulk water, the exposed hydrophilic side-chains are likely to be hydrated, making it more likely to have bridging water than direct hydrogen bonds (Durell and Ben-Naim, 2017).

**FIGURE 1:**
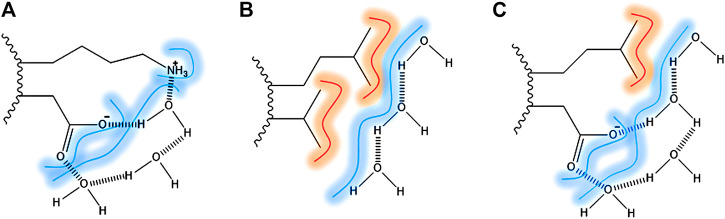
Surface direct forces: amino acid residues exposed to the surface bridged by hydrogen bonds and VDW interaction with water (dash). **(A)** Lysine and aspartic acid as an example of two polar amino-acid side chains bridged by water molecules; **(B)** Leucine and alanine side-chains as an example of two apolar amino-acid side chains and the VDW interactions; **(C)** Leucine and aspartic acid as an example of apolar and polar amino-acid side chains.

The contribution of hydrogen bonds is pivotal for the stabilization of the secondary structure elements. However, it is seen as small for the tertiary and quaternary structure stabilization, due to the presence in similar amounts in the folded and unfolded state. The contribution of water in protein stabilization by forming bridges between protein surface and hydrophilic residues has been raised as significant by many authors. In Figure 1, it is shown the water bridging at the protein surface (Ben-Naim, 2013; Durell and Ben-Naim, 2017). In Figure 1A, the water is bridging two polar side-chains through a hydrogen bond. In Figure 1B, water is shielding two hydrophobic residues exposed by the solvent, through hydrogen bonds among water molecules and VDW interactions between water and the aliphatic chains. In Figure 1C, the water is bridging hydrophobic and hydrophilic residues.

## Are Solvent-Induced Surface Forces Underestimated?

There is not a clear answer to this question. As briefly described earlier, there is a vast literature, using mainly computational prediction, which suggests that the answer is yes. It is not our intention to give a final answer but rather discuss experimental results in which these hydrophilic surface forces play important roles. We will present experimental results of i) protein dynamics in water cavities, ii) the structure and dynamics of core-less proteins, such as plant defensins and toxins, iii) the importance of surface forces in transient interactions in the modulation of multi-domain proteins dynamics and iv) how these forces may modulate reactivity at the surface.

### Methods Available to Study Protein Solvation

One of the methods to study protein solvation is x-ray crystallography, where immobile and symmetric crystal waters can be directly observed in the electron density map. This is important to give information on tightly bound waters, which frequently are involved in catalysis and biological function.

The Nuclear Magnetic Resonance (NMR) spectroscopy can also be used, allowing the measurements of protein hydration in solution. The residence time obtained for the interior water molecules is in the range of 10^−8^ to 10^−2^ s, while surface water molecules are in the sub nanoseconds timescale. For this, NMR is a powerful technique to obtain information on the location and the residence time of individual hydration water molecules, being able to distinguish between tightly bound and unbound waters from the solvation layer. As mentioned before, it is an important mechanism involved in protein stability.

The method is based on the dipolar coupling between the hydrogen nuclei spin of water and hydrogens of the protein. This is achieved by measuring the nuclear Overhauser effect at the laboratory frame (NOE) and the rotating frame (ROE). Otting and collaborators (Otting et al., 1991b) have shown the difference in the residence time between water molecules in the protein interior as well as in the protein surface and compared with the water molecules observed in the corresponding crystal structure. When short mixing times are used in the NOESY/ROESY experiments, the contributions from auto relaxation and spin diffusion are minimal. As a result, the NOE/ROE intensities are almost exclusively from the contribution of cross-relaxation between water-protein nuclear spin. The authors constructed protein hydration models to verify how the sign and value of the NOE/ROE intensity ratio inform about the residence time and location. A negative NOE/ROE intensity ratio reveals dipolar interaction between the water and the protein residue and the presence of motional retarded water molecules.

It was verified later that non-local water molecules in fast exchange with the interacting water molecule also contribute to the negative ratio (Modig et al., 2004). Therefore, the presence of a negative ratio is not in itself conclusive regarding the presence of local tightly bound water. Thousands of non-local water molecules contribute to the negative NOE/ROE intensity ratio. The non-local effect of this method can be overcome by restricting the water mobility and exchange or the amount of water. This is the case of the water cavity of thioredoxins, where tightly bound water is found in the water cavity, hydrogen-bonded to the buried aspartic acid and these internal water molecules are motionally retarded (Cruzeiro-Silva et al., 2014; Iqbal et al., 2015).

### Solvation Studies in Reverse Micelles

The measurement of protein hydration is not an easy task, once the quantity of water molecules in the solvent is extensive, and because of its fast motion and exchange. NMR experiments may provide the residence time and locality of water interactions through dipolar magnetization exchange of the hydrogens from protein-water interactions. By comparing the nuclear Overhauser effect at the NOE and ROE it can be verified the contributions of dipolar and chemical exchanges (Otting et al., 1991b). However, when the protein is embedded in bulk water there is a non-local effect that contributes to the intensities, leading to misleading data (Modig et al., 2004). Also, hydrogens exchange with the hydrogens from water solvent can make the analyses confusing to be evaluated.

To minimize the difficulty in measure protein hydration by NOE and ROE *via* NMR experiments, Wang has proposed the idea of protein encapsulation through a reverse micelle. It provides a reduction of water molecules in the system, which consequently leads to a decrease in hydrogens exchange and the non-locality artifact. It is also possible to regulate the protein tumbling time using the solvent solution with low viscosity to improve the relaxation parameter and the signal/noise ratio.

Wang et al. used ^15^N, ^2^H-ubiquitin encapsulated in reverse micelles in propane solution. It is remarkable the difference between the two spectra, ubiquitin in aqueous solution and the reverse micelle. The non-local effect and the long-range coupling to water were not present in the spectra, but just NOEs and ROEs with NOE distance from the surface were in the ^1^H planes of ^13^C-resolved NOESY and ROESY, which validating the method. The molecules of water in the protein interior have a residence time from about 10^−8^ to 10^−2^ s while the water molecules from the protein surface hydration in solution present residence time of sub-nanosecond (Otting et al., 1991a). By the *σ*_NOE_/*σ*_ROE_ ratio, it was verified the interaction between the water and the protein, where the ratio goes from 0 to −0.5 (small and high residence time, respectively). The ratio near -0.5 refers to protein-water interaction through the rotational correlation time and the ratio of 0 to interactions with times smaller than the rotational correlation time of the protein.

The NOE and ROE measurements showed to be a powerful method to verify protein hydration through NMR. Protein hydration has been studied because of its role in macromolecule functions, as in the case of ubiquitin which is an important protein involved in interactions that regulated the protein degradation inside the cell. It has also been reported the involvement of water molecules in protein-protein interactions and the association of protein subunits.

### Core-Less Proteins–A Particular View at the Dynamics of Plant Defensins

There are a vast number of proteins that are stabilized by an extensive number of disulfide bridges. Plant defensins are a good example of these proteins and they share the same cysteine stabilized *αβ* fold (CSαβ) (Thomma et al., 2003; Thevissen et al., 2004). Their primary sequence is diverse, where the cysteines are the only conserved residues required to maintain the CSαβ fold (Gachomo et al., 2012). Similar cysteine stabilized folds are also known such as toxins, channel blockers, disintegrins, and many enzyme inhibitors (Yount and Yeaman, 2004). Different from typical globular proteins, they are core-less globular proteins. They lack a typical hydrophobic core, and instead, they are stabilized by disulfide bonds and the contacts between surface-exposed hydrophobic and hydrophilic residues. Most of the hydrophobic residues are exposed to the protein surface, and yet plant defensins are soluble and monomeric. Surface forces, along with the covalent disulfide bonds are the main forces that stabilize the CSαβ.

**FIGURE 2:**
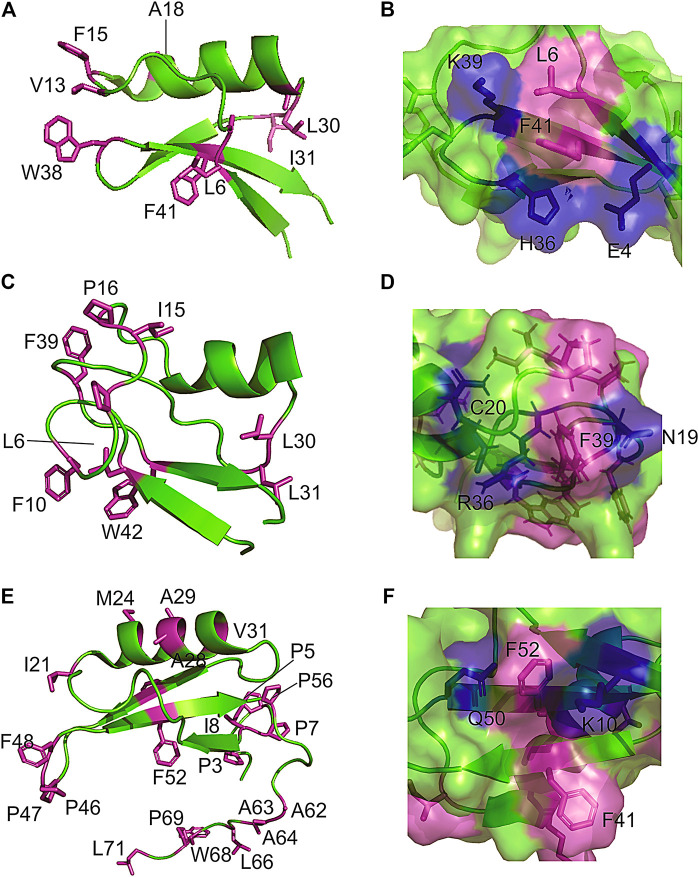
Examples of hydrophobic residues exposing to solvent forming a hydrophobic/hydrophilic surface cluster in plant defensins. The extended and hydrophilic side chains of some amino acids are showing to protect the surface patch in **(A,B)** plant defensins Psd1; **(C,D)** Psd2; and **(E,F)** Sd5.

Because defensins have a high number of disulfide bonds, it was implicit that they would possess restricted backbone mobility. Remarkably, measurements of nuclear spin relaxation by NMR revealed extensive conformational dynamics in regions of the protein that are dominated by exposed hydrophobic residues. For instance, the Psd1 plant defensin exhibits millisecond time scale dynamics in the *β*1/*α*1 loop (Medeiros et al., 2010), which forms the membrane recognition site. Almost all hydrophobic side chains in Psd1 are exposed at this surface patch (Figures 2A,B). Similarly, Sd5 defensin undergoes millisecond dynamics involving all secondary structure elements (de Paula et al., 2011; Machado et al., 2018b). For this defensin, hydrophobic residues are exposed all over the protein surface (Figures 2E,F). Another example of this feature is found in Psd2, where the presence of conformational exchange is correlated with the exposure of hydrophobic residues at the protein surface (Figures 2C,D).

A closer look at the structure in the solution of plant defensins, along with the analysis of the contact map provided by the nuclear spin dipolar interactions (NOEs) used in the structure calculation, points toward the formation of surface clusters. Interestingly, the exposed hydrophobic residues form dynamic hydrophobic/hydrophilic surface clusters containing long and linear extended polar side chains, such as arginine, lysine, and glutamate. Prolines are also present in such clusters, conferring some hydrophilic/hydrophobic balance to the surface-clusters. These hydrophobic surface clusters showed in Figure 2 for the plant defensin Psd1, Psd2, and Sd5 (Machado et al., 2018c), align with the idea that water solvation can bridge surface polar and apolar side chains (Ben-Naim, 2013, 2014), acting as a surface direct stabilizing force (Figure 1).

For plant defensins, long polar side chains interact with the surface hydrophobic amino acid side chains, minimizing their exposure to the solvent. It protects the hydrophobic amino acids from complete exposure to the solvent, making cysteine stabilized folds water-soluble and monomeric.

Measurements of protein dynamics by NMR, verifying the presence of conformational exchange at the hydrophobic surface clusters, are a powerful tool to study these clusters. The measurement of ^15^N relaxation parameters (R1, R2, and hetero-nuclear NOE) enables the mapping of the residues in thermal conformational flexibility (pico- to nanosecond timescale dynamics) and in conformational exchange (micro- to millisecond dynamics) at the core-less proteins, leading to the description of the backbone dynamics. The measurement of CPMG relaxation dispersion profiles in several temperatures enables the quantification of the micro- to millisecond conformational equilibrium and to understand the structural property of the first thermally accessible high energy conformational state. For Sd5 (Machado et al., 2018c), it suggested that the most important changes of the high-energy conformational state are within the *α*-helix, dismantling some of the hydrophobic surface clusters. Remarkably, this high-energy state is more compact. The protein dynamic is the key factor to fully understand the complexity of the intrinsic structural behavior of the hydrophobic surface clusters and to measure their contribution to the stability of cysteine stabilized fold. Further studies are necessary.

### Transient Surface Interactions in Multidomain Proteins

**FIGURE 3:**
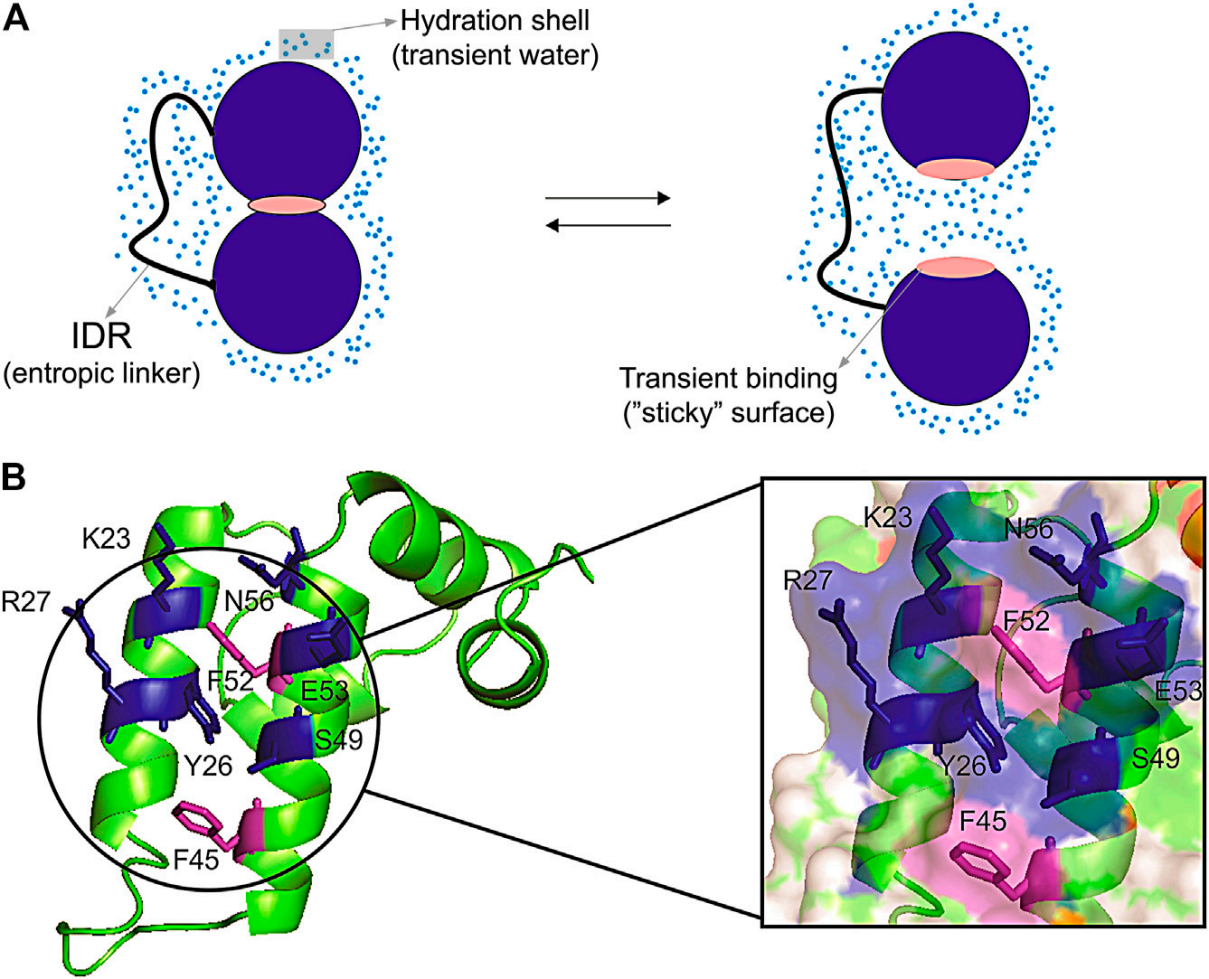
Representation of transient interdomain interactions and surface cluster patch. **(A)** Scheme of a hypothetic open/close equilibrium being regulated through inter-domain transient interaction. The two domains (blue circumference) linked by an intrinsically disordered region (IDR) are interacting in a close conformation through the transient binding of a “sticky” surface (pink), and the solvent-induced interaction in an open conformation. The transient binding sites (“sticky surfaces”) can be formed by defective packing, which may lead to insufficiently dehydrated hydrogen bonds and exposed hydrophobic residues. The blue dots denote the hydration shell formed by transient water molecules; **(B)** The Sis1 J-domain showing the surface patch with exposed hydrophobic residues (purple) protected by polar side chains (blue). This surface patch presents transient inter-domain interactions which are pivotal for protein recognition (Pinheiro et al., 2019).

Surface clusters are not only present in core-less proteins, they are also present in globular proteins and may have a fundamental role in regulating transient interaction in multi-domain proteins (Pinheiro et al., 2019). These transient interactions are difficult to measure and are important to regulate inter-domain motion, which ultimately controls the activity of multi-domain proteins (Figure 3A). Hydration has an important role in mediating these interactions. The force of hydrogen bonds is regulated by the access to water. Fernández and Scheraga (2003) described that the wrapping of the backbone within the protein structure dehydrates and strengthen the hydrogen bonds, while insufficiently dehydrated hydrogen bonds created by unwrapping cause by packing defects make a “sticky” surface and prompt to bind to other sites (Fernández and Scheraga, 2003). Corroborating this idea, binding sites are often involved in conformational exchange (Valente et al., 2006) possibly due to the packing defects, promoting an increase in water access and exposure of hydrophobic surfaces (Figure 3A). Water also mediates protein-protein interfaces, bridging the protein interfaces (Papoian et al., 2003). It has also been proposed that small organic molecular (osmolytes) that can accumulate in the cells modulate thermodynamic stability or proteins, enzyme activity, and protein oligomerization (Rumjanek, 2018). The addition of cosolvents (osmolytes) changes the hydration shell of proteins, modulating the hydrophobic effect (Van Der Vegt and Nayar, 2017). Changes in hydrations also promote allosteric effects, which as measured for hemoglobin (Colombo et al., 1992). Consequently, the hydrophobic surface clusters observed in the core-less protein have the potential to modulate intermolecular and inter-domain interactions.

Multi-domain proteins are abundant in eukaryotic genomes and are advantageous to accelerate the search for cellular targets (Madan et al., 2011; Zmasek and Godzik, 2012). The domains are defined as an evolutionary and independent unit that can be part of a multi-domain protein or even be an independent single protein (Vogel et al., 2004). The domain function can vary, displaying different catalytic activity, cofactor binding, carry protein-protein recognition motifs, and more (Vogel et al., 2004). In evolution, novel domain arrangements are formed, increasing diversity and performance. The creation of new domain architectures has high adaptive potential through evolution and a relevant functional role (GUON and CHUNG, 2016). The inter-domain dynamics in multi-domain and multicomponent proteins is a key feature that determines protein functionality (Valente et al., 2006). The interdomain dynamics are still poorly known, mainly because it involves the understanding, among other features, of the thermodynamic role of the flexible linkers (intrinsically disordered region–IDR), known to contribute to the entropy of binding and allosteric events (entropic linkers) (Wright and Dyson, 2014; Li et al., 2018). It also involves the understanding of the structural features of the linkers and inter-domain surface interaction patches (Zhu et al., 2000; Wriggers et al., 2005). There are only a few examples in the literature that shows that the inter-domain transient interactions, along with the flexible linkers are the key elements that regulate the flexibility of a multi-domain and ultimately the protein function. In this review, we will show some examples of proteins where the flexibility of the domains has been recognized and where the surface interaction patches were described.

A reported example is the inter-domain contacts and stability of Serralysin protease from *Serratia marcescens* (Zhang et al., 2015). This protease family is known to be involved in pneumonia, empyema, urinary tract infection, and more. It presents high similarity, being composed of an N-terminal helix, a protease domain with an active-site motif (HEXXHXXGXXH), and an Asp/Gly-rich Repeats-in-ToXin (RTX) domain in the C-terminal portion. The folding of serralysin protease is regulated through the binding of Ca^2+^ at the C-terminal domain, and interestingly the folding hyper-stabilization is due to domain-domain interactions between the N and C-terminal of RTX protease (Zhang et al., 2015).

Another interesting example is in the Hsp40 co-chaperones family, for which the surface inter-domain interaction patch was recently described and mapped. It regulates the transient surface interactions of the J-domain and shows to be important for protein functions. The importance of the co-chaperones family is widely known in the literature for its activity in protein quality control in the proteostasis system (Summers et al., 2009; Kampinga and Craig, 2010; Cyr and Ramos, 2015). The Sis1 protein, an Hsp40, is composed of an N-terminal J-domain followed by a glycine-rich flexible linker containing G/F and G/M, and a C-terminal domain that contains the dimerization interface. Recently, Pinheiro et al. (2019) showed the pivotal role of transient inter-domain interactions of Sis1 protein. The comparison of the solution structure of the Sis1 J-domain with the full-length protein and its interaction with Hsp70, essential for the delivery of the client protein, revealed a surface interaction patch composed of hydrophobic and positive residues in the helix II and III (Figure 3B). The patch mediates internal transient interactions in the full-length Sis1 and the interaction with Hsp70. The patch is formed by residues V2, T39, F52, D9, R27, and R73 and resembles the hydrophobic surface clusters described for the plant defensins Sd5, Psd1, and Psd2 (Machado et al., 2018c), in which the exposed hydrophobic residues are protected by polar side chains (Figure 3B). These transient inter-domain interactions mediated by the hydrophobic surface cluster are favorable for the Hsp40 and Hsp70 interaction, being pivotal for the delivery of the client protein (Pinheiro et al., 2019).

An important feature in multi-domain architecture is the presence of IDRs, which work as flexible linkers, modulating allosteric events and the kinetics of target recognition (Vuzman and Levy, 2012; Li et al., 2018). The growth factor receptor-bound protein 2 (Grb2) presents a high quantity of loops which are pivotal for the protein dynamics and enable its necessary plasticity to bind to multiple cellular targets (Yuzawa et al., 2001; Sanches et al., 2019, 2021). Grb2 presents an equilibrium between monomeric and dimeric states, which is fundamental for the activation and regulation of signaling pathways (Yuzawa et al., 2001). The flexibility of the monomer of Grb2 is pivotal for the protein function in the recognition of cellular partners (Yuzawa et al., 2001). The SH2 domain of Grb2 comprises a unique dynamic behavior involving two independent subdomains. Subdomain I, responsible for the direct recognition of the phosphotyrosine, is in the fast-exchange regime and subdomain II, is the phosphotyrosine +2 residues specificity pocket is in the intermediate exchange regime (Sanches et al., 2020). This fascinating dancing behavior found in each protein is fundamental for molecular recognition. Further studies are necessary to fully understand the correlation between the inter-domain dynamics and protein function.

Nowadays, the interest in studying and understanding the way of working intrinsically disordered proteins (IDPs) has been increasing. Due to the intrinsic hydrophilicity, IDPs and IDRs are highly hydrated, but they do not behave as a random coil. They are intrinsically disordered, but the remaining order is important for the protein functions, especially when acting as entropic linkers in multi-domain proteins (Wright and Dyson, 2014). The IDPs and multidomain proteins are present in protein-protein interactions in many important biological systems. For example, it is reported that the intrinsically disordered region adopt a folded structure upon binding with its respective partner, or even with high concentrations of osmolytes in cellular stress condition (Rumjanek, 2018). Recently, Borgia and colleagues showed a disordered protein-protein interaction with physiological importance (Borgia et al., 2018). They investigated an interaction between the histone H1 linker, a largely unstructured and positively charged protein, known to be present in chromatin condensation, and the ProT*α* nuclear protein. The ProT*α* is fully disordered, negatively charged, and has an important participation in chromatin remodeling, transcription, cellular proliferation, and apoptosis. When interacting, H1 and ProT*α* remain unstructured, and the binding is driven by the large opposite net charge of the proteins. The interaction between H1 and ProT*α* represents significant evidence that a high-affinity interaction can occur even in the absence of a well-defined binding site. The presence of electrostatic interactions was demonstrated to be enough for the complex stabilization. It is an excellent example of the importance of expanding our studying and understands of the IDP’s functionality.

### Surface Effect on the Reactivity of Certain Protein Post-Translational Modifications, Such as S-Nitrosation

**FIGURE 4:**
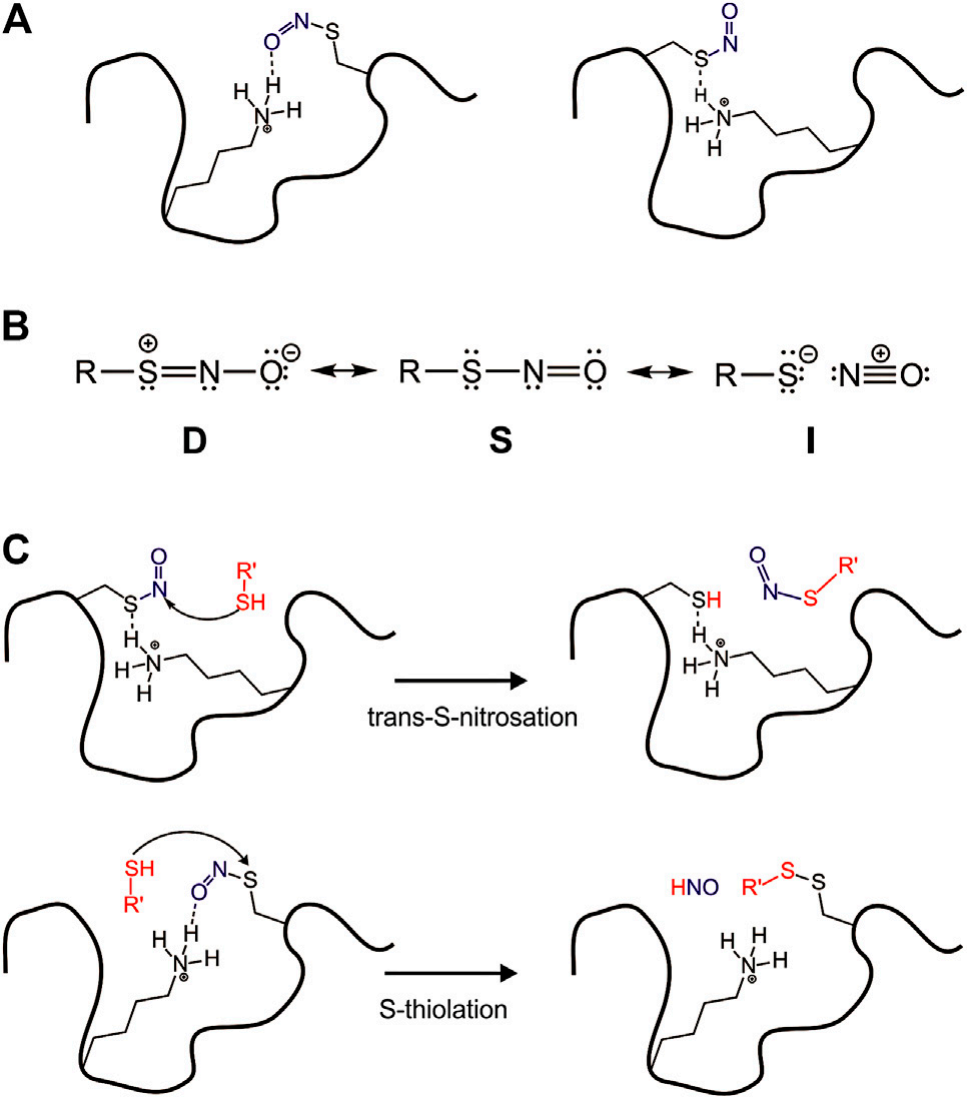
Surface effect on the reactivity of S-nitrosation site. **(A)** Surface interactions modulating–SNO group in the protein; **(B)** RSNO as three resonance structures; **(C)** The protein environment driving nucleophilic attack in different positions in the RSNO.

Nitric oxide (NO) is a free radical known as an important second messenger for signal transduction in cells. In mammals, three nitric oxide synthase isoforms are responsible for NO synthesis: neuronal (nNOS), inducible (iNOS), and endothelial (eNOS) (Smith and Marletta, 2012)^.^ Once formed, NO reacts with the cysteine side chain as well as peptides and proteins that possess cysteine residues in their sequence, forming a posttranslational modification named S-nitrosation. A range of diseases including Parkinson’s, Alzheimer’s, heart failure, arrhythmia, diabetes (type I and type II), asthma, and cancer correlates with dysregulated S-nitrosation of proteins (Anand and Stamler, 2012). Due to dynamics and reversible features, S-nitrosation of protein is also implicated in regulating enzymatic activity, protein stability, subcellular localization, and protein-protein interaction (Morris et al., 2016). Than, we want to discuss how S-nitrosation is a function of the surface interactions (Figure 4A).

In the cell, S-nitrosothiols (RSNOs), the products of S-nitrosation, play an important role due to their capacity to store, transport, and transfer NO to different targets, a biological event known as *trans*-S-nitrosation. The pathway of *trans*-S-nitrosation consists of a nucleophilic attack of a thiolate or thiol at the nitrogen atom from RSNO. Despite the recognized biological importance of RSNOs, the reactivity of these species deserves more investigation to understand how or what exactly controls it *in vivo*. Besides the mechanism of *trans*-S-nitrosation, RSNOs can perform an alternative mechanism of S-thiolation, which consists of a nucleophilic attack of the thiol at the S atom from the–SNO group. That second possible reaction leads to a disulfide formation and HNO release, an nitrogen species with great pharmacological potential (Miranda, 2005).

The mechanisms of *trans*-S-nitrosation and S-thiolation are possible due to RSNOs distinct electronic structure. Timerghazin et al. (2007) have proposed three resonance forms to RSNOs to predict and understand their overall chemical reactivity, structural, and conformational properties. According to this idea, the electronic structure of RSNOs consists of a combination among the conventional RSNOs structure (S), which has a single bond between S and N atoms, a zwitterionic structure (D) with a double bond between S and N atoms, and an RS^−^/NO^+^ ion pair (I) (Figure 4B).

Despite the great number of S-nitrosated proteins identified *in vivo*, the reactivity control of those biological RSNOs is not well understood. To investigate this phenomenon, Talipov et al. have used a range of models to computationally demonstrate that specific interactions of RSNOs with charged and polar residues in proteins can result in significant modification of RSNO characteristics, including their reactivity (Talipov and Timerghazin, 2013; Timerghazin and Talipov, 2013). These reported interactions stabilize and modulate formal charges in RSNOs nitrogen and sulfur atoms, so the protein environment tightly drives a nucleophilic attack in the RSNO (Figure 4C).

The reactivity of cysteine residues to S-nitrosation is dependent on the protonation state of the thiol group. Thiolates are nucleophiles prompt to attack the RSNOs. When the nucleophilic attack is at the sulfur the product is a disulfide (thiolate reaction), conversely, when the attack is at the nitrogen the reaction is S-trans-nitrosation (Talipov and Timerghazin, 2013; Timerghazin and Talipov, 2013). The content of thiolate depends on the pKa of the cysteine residue, which varies according to the amino acid residues in the microenvironment and to the access to water. Turan and Meuwly (2021) showed that the S-nitrosation of myoglobin can increase the density of water molecules closer to the nitrosation site due to the polar NO group, suggesting that the hydration can be modulated by S-nitrosation (Turan and Meuwly, 2021). Protein surfaces have many nuances that may impact the S-nitrosation/thiolation reaction (Figure 4C). The surface forces acting on a cysteine due to the protein vicinity and access of transient water may be key to the regulation of the cysteine thiol/thiolate equilibrium (free cysteine) or to the stabilization of the resonance form (A, B or C, Figure 4B) in an S-nitrosated cysteine. The role of water is unknown and further studies are needed.

## Conclusion

Hydrophobic collapse is considered the dominant driving force in protein folding in globular proteins. There is an increasing investigation on the contribution of surface direct forces to protein stability and folding. The balance between hydrophobicity and hydrophilicity is crucial for protein folding and also for its structural properties. There is no clear answer if the direct surface forces are underestimated. We discussed experimental results on the structure and dynamics of core-less proteins. They lack a hydrophobic core and are stabilized by disulfide bonds and the contacts between surface-exposed hydrophobic and hydrophilic residues. These proteins have many hydrophobic residues exposed to the solvent and yet, they are water-soluble and monomeric. They form locally stabilized hydrophobic surface clusters, in which the hydrophobic side chain is protected by the long linear side chains of the hydrophilic residues. Possibly, the hydration contributes to the cluster stabilization, by forming bridges between hydrophilic and hydrophobic side chains, as illustrated in Figure 1. The measurement of nuclear spin relaxation by NMR was important to describe the hydrophobic surface clusters (Machado et al., 2018c). There is no description in the literature of the stability of these clusters. NMR relaxation and direct solvation studies (NOE/ROE intensity ratio) may contribute significantly to a better understanding of these clusters’ properties and their importance in protein functions.

Surface clusters are also present in globular proteins and may have an important role in regulating transient interaction in multi-domain proteins. CSP analysis through NMR revealed a hydrophobic surface patch in the co-chaperone Hsp40, which modulates inter-domain dynamics, regulates internal transient interaction and the interaction with Hsp70, being pivotal for the protein function.

We also discussed the importance of the protein surface properties to modulate the reactivity of cysteines to the post-translational modification mediated by nitric oxide, forming S-nitrosated species. The methods revised here may be of extreme importance to fully understand the surface effect.
